# Comparable Performance Characteristics of Plasma Thiamine and Erythrocyte Thiamine Diphosphate in Response to Thiamine Fortification in Rural Cambodian Women

**DOI:** 10.3390/nu9070676

**Published:** 2017-06-29

**Authors:** Adrian McCann, Øivind Midttun, Kyly C. Whitfield, Hou Kroeun, Mam Borath, Prak Sophonneary, Per Magne Ueland, Timothy J. Green

**Affiliations:** 1 Bevital AS, Laboratoriebygget Bergen, 5021 Bergen, Norway; Bjorn.Midttun@k2.uib.no; 2 Food, Nutrition, and Health, The University of British Columbia, Vancouver, BC V6T 1Z4, Canada; Kyly.Whitfield@msvu.ca (K.C.W.); tim.green@sahmri.com (T.J.G.); 3 Department of Applied Human Nutrition, Mount Saint Vincent University, Halifax, NS B3M 2J6, Canada; 4 Helen Keller International, Cambodia Country Office, Phnom Penh 12301, Cambodia; hkroeun@hki.org; 5 National Sub-Committee for Food Fortification, Ministry of Planning, Phnom Penh 12000, Cambodia; borathmam@yahoo.com; 6 National Nutrition Programme, Maternal and Child Health Centre, Ministry of Health, Phnom Penh 12202, Cambodia; sophonprak@gmail.com; 7 Department of Clinical Science, University of Bergen, 5021 Bergen, Norway; per.ueland@ikb.uib.no; 8 Laboratory of Clinical Biochemistry, Haukeland University Hospital, 5021 Bergen, Norway; 9 Healthy Mothers, Babies, and Children, South Australian Health and Medical Research Institute, Adelaide 5000, Australia

**Keywords:** thiamine (vitamin B_1_), direct biomarkers, thiamine monophospahte, erythrocyte thiamine diphosphate, plasma

## Abstract

**Background**: Traditionally, vitamin B_1_ status is assessed by a functional test measuring erythrocyte transketolase (ETK) activity or direct measurement of erythrocyte thiamine diphosphate (eThDP) concentration. However, such analyses are logistically challenging, and do not allow assessment of vitamin B_1_ status in plasma/serum samples stored in biobanks. Using a multiplex assay, we evaluated plasma concentrations of thiamine and thiamine monophosphate (TMP), as alternative, convenient measures of vitamin B_1_ status. **Methods**: We investigated the relationships between the established biomarker eThDP and plasma concentrations of thiamine and TMP, and compared the response of these thiamine forms to thiamine fortification using samples from 196 healthy Cambodian women (aged 18–45 years.). eThDP was measured by high performance liquid chromatography with fluorescence detection (HPLC-FLD) and plasma thiamine and TMP by high performance liquid chromatography-tandem mass spectrometry (LC-MS/MS). **Results**: Plasma thiamine and TMP correlated significantly with eThDP at baseline and study-end (*p* < 0.05). Among the fortification groups, the strongest response was observed for plasma thiamine (increased by 266%), while increases in plasma TMP (60%) and eThDP (53%) were comparable. **Conclusions**: Plasma thiamine and TMP correlated positively with eThDP, and all thiamine forms responded significantly to thiamine intervention. Measuring plasma concentrations of thiamine forms is advantageous due to convenient sample handling and capacity to develop low volume, high-throughput, multiplex assays.

## 1. Introduction

Thiamine, also known as vitamin B_1_, is present in the human body as thiamine, thiamine monophosphate (TMP), thiamine diphoshate (ThDP), thiamine triphosphate (TTP), and as the thiamin derivative, adenosine thiamin triphosphate (AThTP) [1]. The biologically active form of thiamine, ThDP, is essential for energy metabolism [1]. Acting as a coenzyme for reactions catalyzed by enzymes such as pyruvate dehydrogenase, α-ketoglutarate dehydrogenase, branched-chain α-ketoacid dehydrogenase complex, and transketolase (TK), ThDP is required for mitochondrial oxidative decarboxylation [2], the pentose phosphate pathway, and the citric acid cycle [3]. Free thiamine and TMP are actively transported into the central nervous system, and are required to maintain sodium and potassium gradients essential for conducting nerve impulses [2,3]. TTP concentrations are regulated by a specific thiamine triphosphatase [4]; however, the role of TTP is not fully understood. Similarly, understanding of the biochemical mechanisms underlying AThTP synthesis and degradation is incomplete [5]. 

Whole-grain cereals, meat, (especially pork), beans, lentils, nuts, fish, and yeast are rich sources of thiamine [6]. However, most of the thiamine in grains is removed when the bran and husk are removed, such as when brown rice is milled to white rice. Overcooking of food may also result in loss of the vitamin. While thiamine deficiency is uncommon in economically developed regions due to diversified diets and thiamine fortification of grains [7,8,9], severe thiamine deficiency leading to beriberi does occur in areas where dietary sources of thiamine are limited, such as Southeast Asia [10,11,12,13]. Thiamine deficiency is also common among alcoholics, leading to Wernicke-Korsakoff syndrome, potentially causing death [14]. Patients with eating disorders [15], cancer [16], acquired immunodeficiency syndrome [17], persistent diarrhea or vomiting [18], or those using drugs such as loop diuretics, penicillins, fluoroquinolones and sulfonamide derivatives can also present with thiamine deficiency [3].

Traditionally, assessment of thiamine status has focused on intracellular concentrations. Approximately 90% of the total thiamine in whole blood is contained within the erythrocytes and leukocytes, predominately in the form of ThDP [19]. The standard functional test is based on the measurement of erythrocyte transketolase (ETK) activity and its relative increase by in vitro addition of ThDP, i.e., the activity coefficient (AC). The test is thought to be the most reliable method for assessing vitamin B_1_ deficiency because erythrocytes are among the first cells to be affected in states of thiamine deficiency [2] and rates of decline in erythrocyte thiamine content parallel those of other major tissues and organs [20]. However, due to various methodological problems including standardization difficulties, pre-analytical sample preparation challenges and sample stability concerns [21], direct methods for assessing thiamine status, focused on thiamine, TMP, total thiamine (thiamine + TMP), ThDP, and TTP in plasma, erythrocytes, whole blood and urine have been developed and are more commonly employed [19,22,23,24,25]. 

Of the direct methods, measurement of erythrocyte (e)ThDP has been favored as it correlates well with the ETK-AC assay [19], and is regarded as a sensitive, specific, and precise method for determining nutritional thiamine status. However, HPLC methods for measuring eThDP are hindered by the absence of standardization between different methods and laboratories, lack of appropriate internal standards, and time-consuming erythrocyte sample preparation, especially in field situations [26]. In plasma or serum, thiamine is typically found in its free form, bound to albumin, or as TMP [5], and a study of rats on a thiamine deficient diet has demonstrated that red cell and plasma thiamine decreased in a similar pattern [27]. However, it has not been conclusively established whether a change in eThDP is a more sensitive marker of thiamine status than changes in plasma thiamine or TMP [19,21,25,28,29,30]. A further drawback of the TK and eThDP based methods is the requirement for washed red cells which are not widely available in most biobanks. Thus, the ability to assess thiamine status using plasma samples by low-volume, high-throughput assays capable of simultaneous measurement of other micronutrients delivers clear advantages. 

Utilizing samples from a thiamine fortification trial [31], the aim of the present laboratory-based work was to determine whether plasma concentrations of the thiamine forms, free thiamine and TMP, reflected thiamine status in humans as measured by the established biomarker, eThDP. 

## 2. Methods

### 2.1. Study Design

This work utilized non-fasting venous samples from a previously published community-based, double-blind, 3 parallel-arm randomized controlled thiamine fortification trial undertaken in Prey Veng province, Cambodia (ClinicalTrials.gov Identifier NCT02221063), described in detail elsewhere [31]. In brief, healthy, non-pregnant, non-lactating Cambodian women (aged 18–45 years) were randomized to receive one of three thiamine-fortified fish sauce formulations: a placebo sauce (control; *n* = 66), low concentration (*n* = 66), or high concentration (*n* = 64) for six months. Before study recruitment, a master identification list was created using online randomization software (GraphPad Software Inc., La Jolla, CA, USA) to allow for nonstratified, individual, equal randomization to three study arms. Cambodian field staff then assigned identification numbers sequentially during baseline data collection. Exclusion criteria included; villages participating in other nongovernmental nutrition related programs, and women who were pregnant or lactating, taking a thiamine-containing supplement, planned to leave their village (i.e., for work) during the six month study period, or if they did not agree to exclusively use the study fish sauce. Study fish sauce was produced by Leang Leng Enterprises (Phnom Penh, Cambodia), with placebo (control) sauce containing no detectable thiamine, and thiamine-fortified fish sauce containing a low (2 g/L) or high (8 g/L thiamine hydrochloride (≥98% purity), Huazhong Pharmaceutical Co., XiangYang, China) concentration of thiamine. Women received their first supply of fish sauce after baseline blood collection, then every two weeks thereafter during household visits from field staff. Women were instructed to consume the study fish sauce *ad libitum* as they normally would.

All women provided informed written consent and ethical approval was obtained from the National Ethics Committee for Health Research in Cambodia (0245NECHR; 386NECHR) and the University of British Columbia Clinical Research Ethics Board in Canada (H14-00103).

### 2.2. Date and Blood Collection

Demographic information and blood samples were collected at baseline (6–17 October 2014) and post-intervention (six months later; 22–29 April 2015). Non-fasting venous blood samples were collected into evacuated EDTA-containing tubes (Vacutainer, Becton Dickinson; Mississauga, ON, Canada) from each woman. Blood samples were stored on ice, and transported within 5 h to the National Institute for Public Health (NIPH) laboratories in Phnom Penh for processing. Blood samples were centrifuged at 3000 rpm for 15 min at 4 °C, and plasma and buffy coat were removed. Erythrocytes were washed three times with phosphate-buffered saline (Amresco; Solon, OH, USA), divided into aliquots and stored at −80 °C. Baseline and post-intervention samples were batch shipped on dry ice to the University of British Columbia (UBC) in Vancouver, Canada in June 2015. Plasma samples were shipped on dry ice from UBC to Bevital AS, Bergen, Norway in October 2015. 

### 2.3. Biochemical Analyses

eThDP was measured at UBC using reverse-phase high performance liquid chromatography with fluorescence detection (HPLC-FLD) according to Lu & Frank (2008) [25], with modifications. In brief, previously frozen packed erythrocytes were vigorously vortex mixed with deionized water and 10% wt/vol trichloroacetic acid in deionized water, placed on ice for 15 min, and then centrifuged (13,000 *g*, 10 min). An aliquot of supernatant was then washed twice with water saturated methyl-tert-butyl ether, before a 150 μL aliquot from the aqueous layer, was transferred to a 96 well plate. HPLC analysis was performed using an Agilent 1260 Infinity system with a Poroshell 120 EC-C18 column, 3 × 50 mm, 2.7 μm, protected by a Poroshell FastGuard C18 precolumn (Agilent Technologies, Santa Clara, CA, USA). Quantitation of eThDP was based on peak area and external calibration using ThDP calibration solutions (Sigma-Aldrich, Oakville, ON, Canada), range ~20–800 nmol/L. Recoveries of low (16.8 nmol/L) and high (42.1 nmol/L) standards in deionized water were 102.5% and 93.2%, respectively. Between-day CVs were <9%.

Thiamine and TMP, the prevailing forms in plasma [5], were measured at Bevital AS (www.bevital.no, Bergen, Norway) by adding these thiamine forms to an existing low-volume, high-throughput, multiplex assay capable of measuring biomarkers related to B-vitamin status (B2, B6), tryptophan metabolism and inflammation in human plasma [32]. Briefly, deproteinized plasma containing isotope-labelled internal standards (100 nmol/L of ^13^C_4_thiamine and ^2^H_3_TMP) was injected into a Zorbax stable-bond C8 reversed-phase column (150 × 4.6 mm, particle size 3.5 m) equipped with a Zorbax stable-bond C8 reversed-phase guard column (12.5 × 4.6 mm, particle size 5 µm), mounted in an Agilent series 1100 HPLC system, coupled to an API 4000 triple-quadrupole tandem mass spectrometer from Sciex (Ontario, Canada) with electrospray ionization (ESI) source operating in positive ionization mode. The method quantified thiamine and TMP by including the ion pairs 265.0/122.0 (quantifier) and 265.0/144.0 (qualifier) for thiamine, and 345.0/122.0 (quantifier) and 345.0/224.0 (qualifier) for TMP. Ion pairs for the internal standards were 269.0/122.0 (quantifier) and 269.0/148.0 (qualifier) for ^13^C_4_thiamine, and 348.0/125.0 (quantifier) and 348.0/224.0 (qualifier) for ^2^H_3_TMP. Thiamine and TMP were purchased from Sigma-Aldrich, and the isotope labeled internal standard ^13^C_4_-thiamine was from Sigma-Aldrich while ^2^H_3_-TMP was from Buchem (Apeldoorn, The Netherlands). Analytes and internal standards were all of purity >99%.

### 2.4. Statistical Analysis 

Geometric means with 5th and 95th percentiles are reported. Correlation analyses were performed using Spearman coefficients. Responses to intervention were examined by one-way analysis of variance (ANOVA) with change (baseline—post-intervention values) as the dependent variable, treatment group (control, low, high) as the factor variable, and Tukey post-hoc test to control for multiple comparisons. Variation in response explained by the intervention was examined by multivariate general linear model (GLM), and indicated by adjusted R-squared. *p* value ≤ 0.05 was considered statistically significant. SPSS version 23.0 (IBM Corp., New York, NY, USA) was used for all statistical analyses.

## 3. Results

### 3.1. Performance of the LC-MS/MS Assay for Plasma Thiamine and TMP

The ion-pairs used for detection were 265.0/122.0 for thiamine, 345.0/122.0 for TMP, 269.0/122.0 for ^13^C_4_-thiamine, and 348.0/125.0 for ^2^H_3_-TMP. Retention times were 4.58 min for thiamine and ^13^C_4_-thiamine and 2.84 min for TMP and ^2^H_3_-TMP. The limit of detection for thiamine and TMP was <0.25 nmol/L and 0.46 nmol/L, respectively. Within-day CV was 4% for thiamine and 8% for TMP. Between-day CV was 7% for thiamine, and 16% for TMP. Other specific details of the assay have been published previously [32]. 

### 3.2. Population Characteristics and Concentration of Thiamine Forms at Baseline

The study population consisted of 196 women with baseline characteristics described in Table 1. At baseline, the plasma concentrations of thiamine, TMP, and plasma total thiamine correlated significantly with eThDP (*r* = 0.41–0.57, *p* ≤ 0.01; Table 2). 

### 3.3. Responses to Intervention

**Figure 1:**
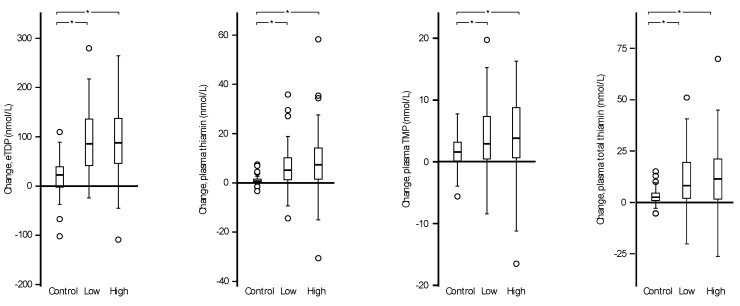
Changes in thiamine forms among individuals randomized to receive placebo (control), low (low concentration (2 g/L)), or high (high concentration (8 g/L)) thiamine-fortified fish sauce. eThDP, plasma thiamine, plasma TMP, and plasma total thiamine responses expressed as change (post-intervention—baseline). Solid horizontal lines indicate median change, the box represents the interquartile range (IQR), the whiskers represent 1.5 times the IQR, and circles indicate outliers. eThDP, erythrocyte thiamine diphosphate; TMP, thiamine monophosphate. Between group differences indicated by, * *p* ≤ 0.01.

Concentrations of the thiamine forms at baseline and post-intervention for each treatment arm are shown in Table 3. eThDP and all the plasma thiamine forms (thiamine, TMP, and total thiamine) increased significantly within the two intervention groups given thiamine-fortified fish sauce (low and high). All thiamine forms also increased within the control group; however, the magnitude of increase was greater in those receiving fortified fish sauce (Table 3 and Figure 1). 

The mean response in the investigated vitamin B_1_ forms within the two intervention groups given thiamine were not significantly different (*p* > 0.33), but the changes in the intervention groups were significantly greater than that observed in the control group (*p* ≤ 0.004). The average change in the fortified groups for eThDP, plasma thiamine, plasma TMP and total thiamine were 53%, 266%, 60% and 117%, respectively, whereas in the control group, the corresponding numbers were 12%, 143%, 52% and 70%. In the multivariate models, the variance explained by the intervention (control sauce or thiamine-fortified sauce) was 39% for eThDP, 45% for plasma thiamine, 11% for TMP, ands 39% for plasma total thiamine (all *p* ≤ 0.001). 

Following the six month intervention, plasma thiamine forms remained strongly correlated with eThDP (ranging from *r* = 0.53–0.61, *p* ≤ 0.01) (Table 4). 

## 4. Discussion

The present study aimed to compare plasma concentrations of thiamine and thiamine monophosphate (TMP) to the established thiamine status indicator, erythrocyte thiamine diphosphate (eThDP). Using samples from a thiamine fortification study among rural Cambodian women, we observed that plasma thiamine, plasma TMP and plasma total thiamine correlated with eThDP at baseline and post-intervention, and that both plasma and erythrocyte concentration of thiamine forms increased significantly following the six month intervention. Of the thiamine forms investigated in the present study, plasma thiamine was the most responsive measure of increased thiamine intake.

### 4.1. Strengths and Limitations

A particular strength of the present study was the measurement of the established thiamine biomarker, eThDP, and the utilization of samples from a 6-month thiamine intervention study, allowing the investigation of a wide concentration range of vitamin B_1_ over time. The low limits of detection of the novel plasma assay allowed quantification of both thiamine and TMP in all the investigated plasma samples. The addition of these vitamin B_1_ forms to a previously validated low-volume, high-throughput, multiplex assay [32] enables comprehensive evaluation of B-vitamin status, involving simultaneous sample processing and analytical procedures. 

Limitations of the study include a lack of dietary data, and the fact that participants were non-fasting at the time of blood collection, thus the effect of acute thiamine intake on plasma thiamine forms cannot be fully evaluated. Participants were not thiamine deficient nor did the original intervention target individuals at highest risk of poor thiamine status. The lower thiamine status of the control group compared to the treatment groups at baseline, suggests the randomization by chance was not optimal. The high variability of response suggests that consumption of the intervention fish sauce as well as intake of dietary sources of thiamine differed considerably within and between each group, however, as with the suboptimal randomization this was not detrimental to the present work. The significant increases in thiamine forms in the control group were consistent with expected changes in background seasonal status and/or food availability [33].

### 4.2. Erythrocyte and Plasma Thiamine Forms as Markers of Thiamine Status

Various methodological problems associated with the standard functional test of thiamine status, measurement of erythrocyte transketolase (ETK) activity [19,21], and advancement in laboratory techniques and instrumentation have facilitated the development of a number of direct HPLC-based methods measuring thiamine, TMP, total thiamine (thiamine + TMP), ThDP and TTP in plasma, erythrocytes, whole blood and urine [19,21,22,23,25]. Of these methods, eThDP is regarded as a more sensitive, specific, and precise method for determining nutritional thiamine status in comparison to the ETK-AC assay [19]. 

In the present study, eThDP concentrations were similar to the levels observed in the limited literature available for healthy, vitamin B_1_-replete women of childbearing age. In a recent study of 51 healthy women of childbearing age (20–45 years) from Vancouver, Canada, mean eThDP was 179 (range: 94–272) nmol/L [34]. Our results for plasma thiamine and TMP were also comparable to the limited, pertinent published data [5,24,35]. Caution has been urged when assessing thiamine status based on plasma measurements as plasma levels account for less than 10% of total blood thiamine [19]. Importantly, the plasma concentration of thiamine forms correlated well with eThDP, at baseline and post-intervention (Table 2 and Table 4). Notably, earlier work has shown that plasma thiamine responds to oral supplementation with vitamin B_1_ [36,37], and Coats and colleagues [35] have previously demonstrated changes in plasma thiamine levels in healthy Cambodian mothers (aged 21–35 years), after receiving oral thiamine hydrochloride (100 mg for five days).

The thiamine field is impaired by a lack of clinically meaningful cut-offs for sufficient thiamine status. Among the four vitamin B_1_ measures investigated in the present study, plasma thiamine was the most responsive of the thiamine forms. Although the variability in response was high, an observation previously reported in a very small study [37], the largest fraction of the variation (45%) in vitamin B_1_ forms explained by the intervention was for plasma thiamine (Table 3). The intervention also explained 39% of the variance for eThDP and plasma total thiamine (thiamine + TMP). The intervention explained only 11% of the variance for TMP suggesting factors other than thiamine intake alone might determine plasma TMP concentrations. Of the thiamine forms investigated in the present study, TMP may thus be the least suitable for assessment of thiamine intake and status, which may also reduce the suitability of plasma total thiamine compared to plasma thiamine alone. The similar explained variances for eThDP and plasma thiamine suggest these two thiamine forms may be equally useful markers of thiamine status. This conclusion is supported by Tallaksen and collegues [37] who reported thiamine increases in whole blood mirrored increases in plasma thiamine.

Healthy adults have an estimated total body thiamine concentration of 25–30 mg [38], however, due to rapid metabolism and turnover of thiamine, and an estimated biological half-life of 9–18 days [39,40,41], routine dietary intake is necessary [42]. Given the limited body reserves of thiamine and its short biological half-life, coupled with the similar response of plasma thiamine to the established marker eThDP, methods capable of quickly evaluating thiamine status using more readily accessible plasma thiamine measures provide obvious advantages. Possible factors underlying the high variability in plasma thiamine levels observed in the present study, such as fasting status and amount of thiaminase in the diet, should be further investigated. 

### 4.3. Thiamine Forms and Compartments

Thiamine homeostasis remains poorly understood and many uncertainties surround the expression and regulation of thiamine transporters, specific thiamine kinases and phosphates, and the distribution of thiamine and thiamine derivatives in different organs and tissues [6]. Limited body stores and a short half-life suggest that six months of intervention was sufficient to achieve steady state. However, thiamine intake was highly variable; women in the low and high groups consumed a mean (SD) of 36 (42) and 127 (153) mg thiamin/day from the fortified fish sauce [31]. The increase in thiamine forms did not differ significantly between the low and high intervention groups. This suggests consumption of fish sauce containing more than 2 g/L thiamine did not confer higher plasma thiamine, plasma TMP, total plasma thiamine or eThDP concentrations. The absence of greater increases in the thiamine forms following consumption of fish sauce containing more than 2 g/L thiamine could also be explained by the large volume of fish sauce consumed by participants throughout the study, as discussed previously [31]. For instance, detailed intake records from a subset of participants indicated that women in the high group could have received 0 mg to 626 mg thiamine in one day from fish sauce alone. This not only suggests the high variability in response could be due to the large variation in intake, but it also supports previous findings which suggest high doses of thiamine (greater than approximately 2.5–5 mg) remain largely unabsorbed in healthy adults [43]. 

Variation in response may also be explained by possible mechanisms related to saturation of transporters or enzymes involved in thiamine uptake or metabolism. Preservation of TMP and eThDP concentrations within a relatively narrow range may reflect that the distribution and turnover of thiamine and the phosphorylated forms of thiamine is driven or limited by the activity of specific enzymes, such as thiamine transporter-1 (THTR-1), reduced folate carrier-1 (RFC-1), thiamine pyrophosphokinase-1 (TPK1) and thiamine pyrophosphatase (TPPase), as well as apoenzyme saturation [42,44,45]. In comparison, the large increases in plasma thiamine concentrations observed in this work further support plasma thiamine as a responsive measure of thiamine intake.

## 5. Conclusions

In the present study, plasma thiamine correlated strongly with the established thiamine biomarker, eThDP, before and after thiamine intervention. Despite high variability in response, the fraction of the variation explained by the intervention was similar for plasma thiamine and eThDP. With performance characteristics similar to eThDP, the use of plasma thiamine as a measure of thiamine intake and status is advantageous because, unlike erythrocytes, plasma or serum is widely available in most biobanks, can be analyzed using multiplex techniques, and is not hindered by logistically complicated sample collection and processing procedures. Future research should investigate the sensitivity, specificity and within-subject reproducibility of plasma thiamine, TMP and plasma total thiamine under conditions of thiamine-depletion and thiamine-deficiency.

## Figures and Tables

**Table 1:** Population characteristics at baseline.

	*n*	Geometric Mean (5th, 95th Percentile)
Age (years)	196	29.5 (21.0, 42.0)
BMI (kg/m^2^)	196	21.7 (17.7, 28.8)
Hemoglobin (g/L)	196	123 (106, 142)
eThDP (nmol/L)	196	159 (87, 248)
Plasma thiamine (nmol/L)	196	1.6 (0.3, 14.0)
Plasma TMP (nmol/L)	188	5.4 (1.8, 17.0)
Plasma total thiamine (nmol/L)	196	7.2 (2.2, 28.5)

BMI, body mass index; eThDP, erythrocyte thiamine diphosphate; TMP, thiamine monophosphate.

**Table 2:** Spearman correlation coefficients for measures of thiamine status at baseline (*n* = 196).

	eThDP	Plasma Thiamine	Plasma TMP ^a^
Plasma thiamine	0.41 **		
Plasma TMP ^a^	0.57 **	0.86 **	
Plasma total thiamine	0.54 **	0.90 **	0.98 **

** *p* ≤ 0.01; ^a^ *n* = 188; eThDP, erythrocyte thiamine diphosphate; TMP, thiamine monophosphate.

**Table 3:** Concentrations of thiamine forms before and after six-month intervention with thiamine-fortified and unfortified (control) fish sauce.

	Control (*n* = 66)			Low (*n* = 66)			High (*n* = 64)				
Thiamine Form	Before	After	Change ^a^	Before	After	Change ^a^	Before	After	Change ^a^	*p* ^b^	*R*^2^
eThDP (nmol/L)	148 (89, 232)	166 (102, 285)	1.12	159 (83, 243)	245 (137, 391)	1.54 ^†^	170 (83, 285)	259 (126, 402)	1.52 ^†^	≤0.001	0.39
Plasma thiamine (nmol/L)	0.7 (0.3, 2.0)	1.7 (0.8, 4.9)	2.43	2.2 (0.4, 14.5)	7.8 (1.3, 28.9)	3.55 ^†^	2.8 (0.4, 17.9)	10.5 (1.8, 49.2)	3.75 ^†^	≤0.001	0.45
Plasma TMP (nmol/L)	3.3 (1.4, 7.3)	5.0 (2.6, 11.3)	1.52	6.5 (2.0, 17.1)	10.6 (4.4, 22.1)	1.63 ^†^	7.4 (2.0, 21.7)	11.7 (5.8, 24.7)	1.58 ^†^	≤0.004	0.11
Plasma total thiamine (nmol/L)	4.0 (1.9, 8.6)	6.8 (3.6, 15.9)	1.70	8.9 (2.8, 28.8)	19.2 (6.6, 48.1)	2.16 ^†^	10.7 (2.4, 46.6)	23.4 (7.5, 72.5)	2.19 ^†^	≤0.001	0.39

Concentration data are geometric mean (5th, 95th percentile); Randomization to treatment arms: control (fish sauce containing no added thiamine hydrochloride), low (low concentration: 2 g/L thiamine), or high (high concentration: 8 g/L thiamine-fortified) group. eThDP, erythrocyte thiamine diphosphate; TMP, thiamine monophosphate. ^a^ Change (post-intervention—baseline vales) expressed as ratio; ^b^ One-way analysis of variance (ANOVA) was used to investigate statistically significant differences between the mean changes in the three groups, with change (post-intervention—baseline vales) as the dependent variable, treatment as the factor variable, and Tukey post-hoc test to control for multiple comparisons. Significant differences between groups are indicated by: ^†^ versus control. Adjusted *R*^2^ is reported as the percentage of variation in response of thiamine forms that is explained by treatment arm.

**Table 4:** Spearman correlation coefficients for measures of thiamine status post-intervention (*t* = 6 months; *n* = 196).

	eThDP	Plasma Thiamine	Plasma TMP
Plasma thiamine	0.61 **		
Plasma TMP	0.53 **	0.83 **	
Plasma total thiamine	0.60 **	0.96 **	0.93 **

** *p* ≤ 0.01. eThDP, erythrocyte thiamine diphosphate; TMP, thiamine monophosphate.

## Abbreviations

ETK	erythrocyte transketolase
eThDP	erythrocyte thiamine diphosphate
TMP	thiamine monophosphate
HPLC-FLD	high performance liquid chromatography with fluorescence detection
LC-MS/MS	high performance liquid chromatography-tandem mass spectrometry
TTP	thiamine triphosphate
AThTP	adenosine thiamine triphosphate
AC	activity coefficient
ESI	electrospray ionization
ANOVA	analysis of variance
GLM	general linear model
THTR-1	thiamine transporter-1
RFC-1	reduced folate carrier-1
TPK1	thiamine pyrophosphokinase-1
TPPase	thiamine pyrophosphatase
